# Modelling reindeer rut activity using on‐animal acoustic recorders and machine learning

**DOI:** 10.1002/ece3.11479

**Published:** 2024-06-25

**Authors:** Alexander J. Boucher, Robert B. Weladji, Øystein Holand, Jouko Kumpula

**Affiliations:** ^1^ Department of Biology Concordia University Montreal Quebec Canada; ^2^ Department of Animal and Aquacultural Sciences Norwegian University of Life Sciences Ås Norway; ^3^ Natural Resources Institute of Finland (Luke), Reindeer Research Station Helsinki Finland

**Keywords:** convolutional neural network, machine learning, on‐animal acoustic recorder, *Rangifer tarandus*, reindeer, rutting behaviour

## Abstract

For decades, researchers have employed sound to study the biology of wildlife, with the aim to better understand their ecology and behaviour. By utilizing on‐animal recorders to capture audio from freely moving animals, scientists can decipher the vocalizations and glean insights into their behaviour and ecosystem dynamics through advanced signal processing. However, the laborious task of sorting through extensive audio recordings has been a major bottleneck. To expedite this process, researchers have turned to machine learning techniques, specifically neural networks, to streamline the analysis of data. Nevertheless, much of the existing research has focused predominantly on stationary recording devices, overlooking the potential benefits of employing on‐animal recorders in conjunction with machine learning. To showcase the synergy of on‐animal recorders and machine learning, we conducted a study at the Kutuharju research station in Kaamanen, Finland, where the vocalizations of rutting reindeer were recorded during their mating season. By attaching recorders to seven male reindeer during the rutting periods of 2019 and 2020, we trained convolutional neural networks to distinguish reindeer grunts with a 95% accuracy rate. This high level of accuracy allowed us to examine the reindeers' grunting behaviour, revealing patterns indicating that older, heavier males vocalized more compared to their younger, lighter counterparts. The success of this study underscores the potential of on‐animal acoustic recorders coupled with machine learning techniques as powerful tools for wildlife research, hinting at their broader applications with further advancement and optimization.

## Graphical abstract

We tested the utility of on‐animal acoustic recorders for recording animal behaviours and documenting the rutting activity of reindeer through their acoustic repertoires. We used machine learning to process the acoustic recordings of mobile animals wearing recorders. We were able to effectively use convolutional networks to describe the rutting activity of reindeer who were wearing on‐animal acoustic recorder.

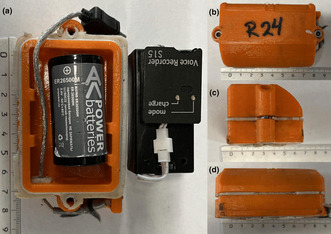

## 1 INTRODUCTION

Acoustic signals facilitate communication and social interaction in animals, and are used for danger avoidance, peer recognition, social learning and mating (Bradbury & Vehrencamp, 2011). Bioacoustics research is advancing our understanding of animal behaviours via the sounds they produce, despite the complexity of interpreting these signals and the early stage of development in this field. Scholars are making headway by tying this information to behavioural ecology, leading to insights into animal occupancy, behaviour and ecology despite technological and methodological challenges (Blumstein et al., 2011; Charlton et al., 2007; Enari et al., 2019; Garcia et al., 2013; Stowell et al., 2017; Studd et al., 2021).

Bioacoustics research spans animal ecology and behaviour, and across ecosystems, researchers use sound to assess community richness and demography (Habib et al., 2007; Laiolo, 2010; Wimmer et al., 2013). Previously, observers or autonomous recording units (ARUs) were used to study these metrics (Shonfield & Bayne, 2017; Wimmer et al., 2013). While ARUs offer improvements over in‐person observers by recording continuously and reducing human bias, their stationary nature means they can miss sounds from distant or mobile animals and struggle with environmental noise interference (Fairbrass et al., 2017; Shonfield & Bayne, 2017). To overcome the limitations of ARUs and better capture animal noises, researchers are increasingly employing on‐animal acoustic recorders to better understand their behaviour and demography.

On‐animal acoustic recorders, commonly used in marine studies, are now being adapted for terrestrial research, offering continuous audio capture of both intentional and environmental sounds from individuals (Ilany et al., 2013; Lynch et al., 2013; Stowell et al., 2017; Studd et al., 2021; Thiebault et al., 2021; Wijers et al., 2018). These devices, custom‐built due to the lack of commercial options for land use, tend to be bulky due to power requirements, difficult to recover and prone to damage by wearers or the environment, affecting the continuity of recordings (Ilany et al., 2013; Lynch et al., 2013; Stowell et al., 2017; Studd et al., 2021; Thiebault et al., 2021; Wijers et al., 2018). Although not yet widespread, early studies show their potential in categorizing events and behaviours (Stowell et al., 2017; Wijers et al., 2018), estimating metabolic costs of sound production (Ilany et al., 2013) and analysing feeding behaviour (Lynch et al., 2013; Studd et al., 2019; Thiebault et al., 2021). However, to date, on‐animal recorders have not been used to study the reproductive activity of terrestrial species.

Processing and analysing the large volume of data produced by these applications remains a significant challenge for researchers. In recent years, collaborations with computer scientists have led to the application of machine learning to bioacoustics (Blumstein et al., 2011; Enari et al., 2019; Stowell et al., 2017; Studd et al., 2021; Thiebault et al., 2021; Wijers et al., 2018). Machine learning, especially convolutional neural networks (CNNs), streamlines data processing and feature identification, outperforming commercial vocalization recognition tools (Dufourq et al., 2022; Knight et al., 2017; Zhong et al., 2020). Despite the effectiveness of CNNs, constructing and training them is complex, lacking clear bioacoustics‐specific guidelines and requiring extensive hyper‐parameter tuning (Dufourq et al., 2022). Transfer learning has emerged as a solution in machine learning to address these challenges (Weiss et al., 2016).

Transfer learning leverages models initially trained on one dataset to make predictions on another, streamlining model selection and reducing hyper‐parameter tuning for bioacoustics researchers (Dufourq et al., 2022; Weiss et al., 2016; Zhong et al., 2020). This method requires fewer annotated examples for training, facilitating faster CNN development (Dufourq et al., 2022). However, most research to date has focused on stationary recorders, with limited application to species wearing recorders (Casoli et al., 2022; Stowell et al., 2017; Studd et al., 2021; Thiebault et al., 2021; Wijers et al., 2018).

Coupling on‐animal recorders with machine learning poses unique challenges, including variable recording quality and increased equipment failure risk. Yet, these methods hold significant potential for understanding animal behaviour and ecology through the lens of emitted sounds (Bravo Sanchez et al., 2021; Ilany et al., 2013; Lynch et al., 2013; Stowell et al., 2017; Studd et al., 2021; Thiebault et al., 2021; Wijers et al., 2018). Despite these benefits, such recorders have yet to be applied to investigate the mating behaviours of terrestrial species.

In the context of reproduction, vocalizations play a crucial role in guiding interactions and sexual selection among terrestrial species, particularly within the family Cervidae (Vannoni et al., 2005). In gregarious species like red deer (*Cervus elaphus*) and reindeer (*Rangifer tarandus*), vocalizations are integral to mate selection, male competition and territory defence, and aid in establishing social hierarchy and regulating sexual selection (Charlton et al., 2007; Espmark, 1964; Garcia et al., 2013; Reby & McComb, 2003a). While red deer vocalizations have been extensively studied, less focus has been directed towards understanding the vocalizations of reindeer (Charlton & Reby, 2011; Reby & McComb, 2003b). In the case of reindeer, vocalizations are used for peer recognition, male‐to‐male competition and territory defence and during the rut, are used to establish harems, with dominant, heavier males gaining access to more females (Espmark, 1964; L'Italien et al., 2012). Consequently, studying male reindeer vocalizations offers the potential to yield insights into mating activity strategies.

Therefore, this study aims to: (1) evaluate the efficacy of on‐animal recorders for studying the rutting activity of male reindeer, (2) develop and train a CNN using transfer learning to identify reindeer vocalizations within continuous recordings, targeting a minimum performance of 90% and (3) describe the grunting patterns of male reindeer during the rut.

## 2 METHODS

### 2.1 Field site and focal individuals

The bioacoustics data were collected at the Kutuharju research site in Kaamanen, Finland (69.1° N, 27.2° E). This site houses a semi‐domesticated reindeer herd within a 45 km^2^ enclosure, comprising about 100 individuals, including calves, females and males. The herd has been under continuous study since 1969.

During the rut (mid‐September to late October), the herd is moved to a smaller pen (Lauluvaara, approximately 13.8 km^2^). At this time, males are weighed and equipped with collars featuring VHF locators for telemetry tracking, as well as acoustics recorders to capture intentional and unintentional sounds. Females are fitted with numbered coloured collars to allow for individual identification. Comprehensive record‐keeping ensures that all animals are of known age and individually recognizable.

Furthermore, parentage analysis is conducted on offspring to determine the reproductive success of each male. It is important to note that for the 2020 sampling period, the parentage of offspring born the following spring had not yet been analysed.

Bioacoustics data were collected during the rutting seasons of 2019 and 2020. In 2019, vocalizations were captured from two males, while in 2020, vocalizations were obtained from six males. However, the sampling time varied from 3 days to 2 months due to equipment issues. Moreover, challenges with the recorders limited our data collection to only seven male reindeer (two from 2019 and five from 2020).

**FIGURE 1:**
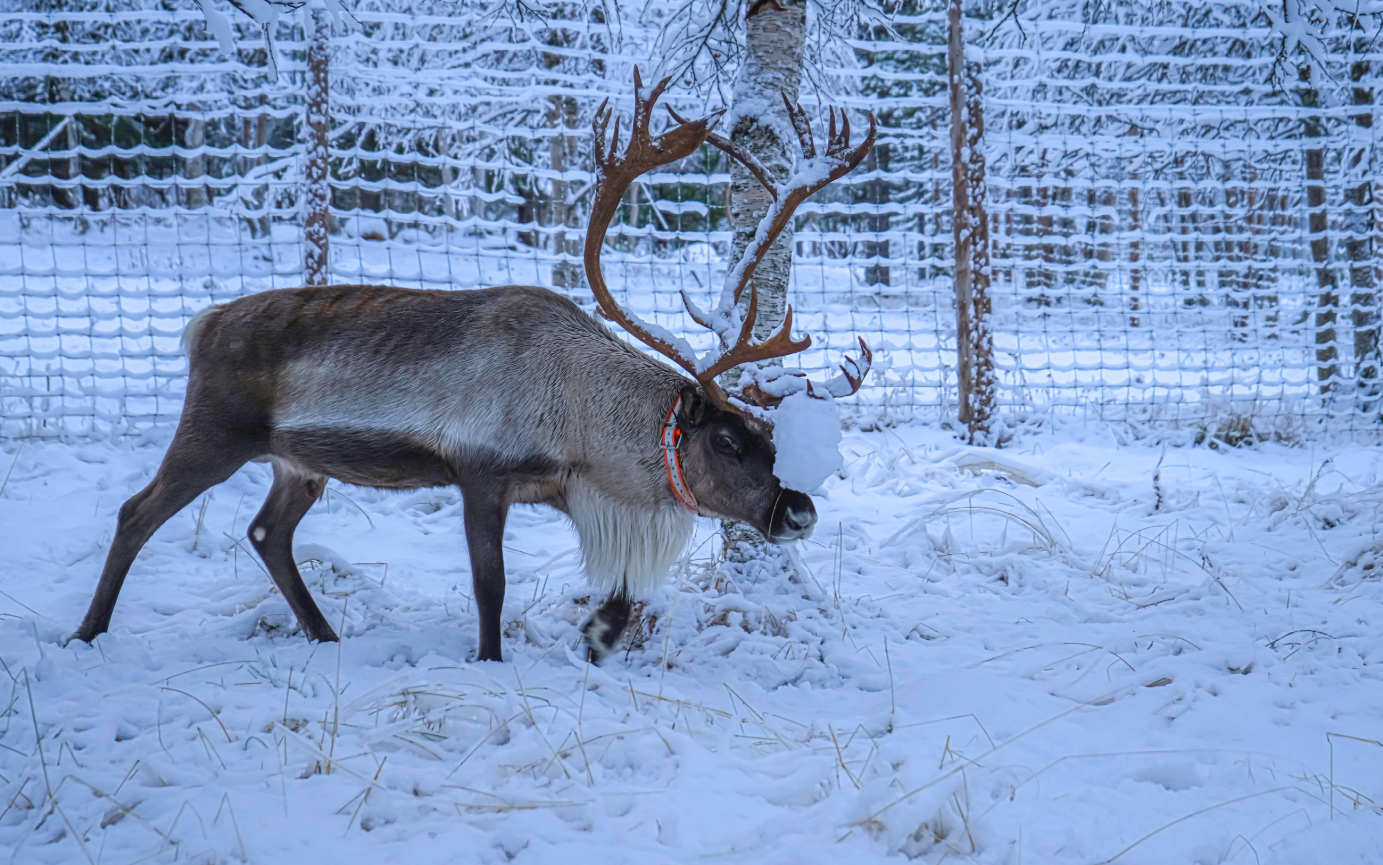
Male reindeer wearing a recorder.

The characteristics of each male are detailed in Table 1, including their distribution across five different age classes (1.5–5.5 years old) and varying weights (Figure 1). Furthermore, reproductive success is indicated by the number of offspring born to a male in the following spring.

**TABLE 1:** Individual reindeer sampling details and physical rut characteristics.

Individual	Age (years)	Year sampled	Recording date start	Recording date end	Number of hourly observations (*n*)	Starting rut weight (kg)	Weight loss at end of rut (kg)	Reproductive success
1.5	1.5	2020	Sept 22	Sept 25	82	83	−5	NA
2.5	2.5	2019	Sept 11	Sept 30	464	96	5	0
3.5–1	3.5	2020	Sept 21	Sept 28	173	100	1	NA
3.5–2	3.5	2020	Sept 22	Oct 9	419	135	30	NA
3.5–3	3.5	2020	Sept 21	Sept 28	164	155	35	NA
4.5	4.5	2020	Sept 21	Sept 30	224	150	39	NA
5.5	5.5	2019	Sept 18	Oct 18	744	140	28	14

*Note*: The 2019 male pre‐rut weights were measured on 11 September 2019, and their post‐rut weights were measured on 26 November 2019. The 2020 male pre‐rut weights were measured on 21 September 2020, and their post‐rut weights were measured on 9 November 2020. Reproductive success denotes the number of offspring born to a male the following spring.

### 2.2 Acoustic recorders and acoustic analysis

**FIGURE 2:**
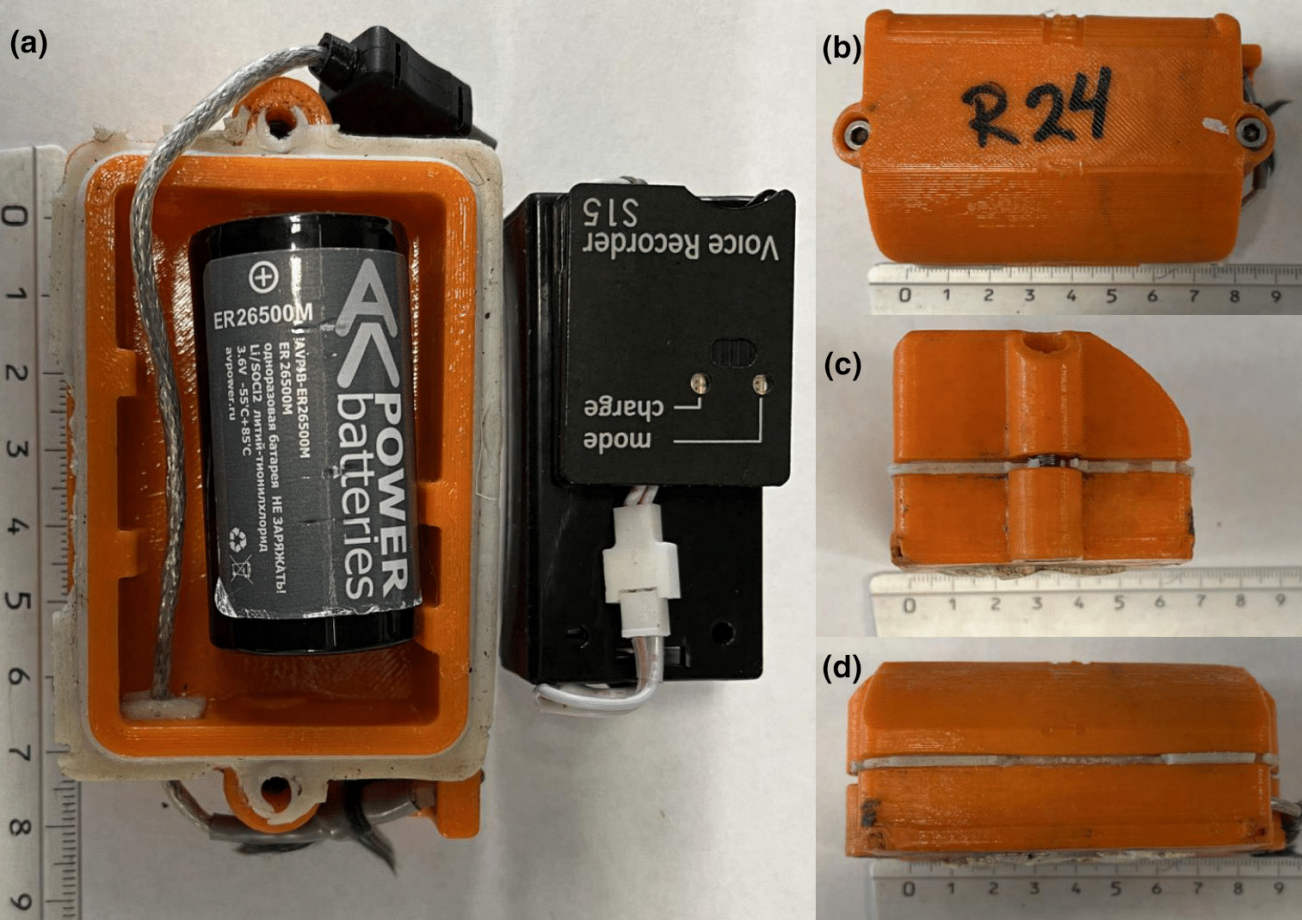
The on‐animal acoustic recorder used to record the rutting vocalizations of the reindeer. The orange case is the 3D‐printed weatherproof housing containing the Soroka‐15E recorder. The four panels represent the (a) interior, (b) top, (c) side and (d) front. The ruler denotes units in centimetres.

During the translocation of the males, they were outfitted with on‐animal acoustic recorders. These devices housed SOROKA‐15E recording units from TS‐Market Ltd., Zelenograd, Russia, capable of capturing the animals' vocalizations with an amplitude resolution of 16 bits and a sampling rate of 16 kHz (Figure 2). The recorders enabled us to obtain continuous audio data throughout the breeding period. For storage purposes, each recorder was equipped with a 256‐gigabyte microSD card, providing the capacity to record over 92 days of audio. Furthermore, a 9000‐milliamp‐hour, 3.6‐volt lithium‐ion battery powered each recorder, ensuring functionality for over 2 months.

The recorder housings provided by TS‐Market Ltd. were fabricated using 3D printing technology. Before deployment on the animals, the recorder batteries were connected and underwent a thorough testing process to ensure functionality. The housing itself, consisting of two halves, was securely sealed with silicone to safeguard the recorders against water damage and then fastened with screws for added integrity. For attachment to each animal's collar, a combination of silicone, metal hose clamps and a protective rubber sheath was utilized, ensuring the recorders remained firmly in place.

Observers documented each male's status and harem during the ruts. Based on data gathered from agonistic interactions among males recorded during the rut, a clear dominance hierarchy was evident, established primarily on win‐lose frequencies. The size of a male's harem was noted throughout these observations. Dominant males, commanding a harem, were given a corresponding label, whereas those without were categorized as subdominant. Fluctuations in dominance among numerous males occurred based on the presence or absence of more dominant males. Dominance during the rut was mainly determined through observations. Observers monitored the males' status for about 20% of the rutting period, providing the basis for audio playbacks. Specific sounds and behaviours were documented during these observed hours, serving as a model to categorize the remaining recordings according to these known patterns. Each hour of each male was analysed through audio playback, with status potentially changing based on displayed behaviours and grunts. Typically, a male's status remained constant for extended periods, with observed shifts from dominant to subdominant and back to dominant occurring as quickly as 3 days. Notably, males did not continuously alternate statuses during these periods, and when a male lost a harem to another male, the displaced male often remained subdominant for an extended period.

In cases where a male was not observed for periods (owing to them occasionally being unlocatable), assessments of their status were conducted during playback of their recordings. For instance, if a playback revealed a male grunting with audible background sounds of females and calves, it was inferred that he held a dominant status. Conversely, the presence of rival male grunts directed at the subject male, if met with silence on the latter's part, suggested his sub‐dominance. If a recording of a male featured no other sounds and he wasn't grunting, it was presumed he was in search of a herd and thus subdominant. Nevertheless, in instances where a male's status was ambiguous during playback and no observational data were available, his status was marked as ‘unknown’. Confirmation of such males' statuses was deferred until more definitive evidence could be obtained.

We employed Sonic Visualiser (Cannam et al., 2010) for the analysis and annotation of the recordings. Each recording underwent annotation using the ‘boxes layer’ feature, enabling the delineation of intentional and unintentional noises via bounding boxes. This allowed for the documentation of start and end times for each sound within the recordings, as well as the minimum and maximum frequency of each vocalization. Notably, a binary label (presence or absence) was assigned to each bounding box. For the presence class, bounding boxes were strategically positioned around individual and repeating vocalizations to capture the grunting behaviours of the reindeer. Incidental calls from other reindeer were intentionally left unannotated to prevent an excessive impact on the CNN training due to the over‐sampling of activities of the focal individuals. Bounding boxes of varying lengths were placed and utilized to annotate the absence class throughout each recording, ensuring comprehensive CNN training by encompassing a wide range of unintentional sounds (biophony, geophony and anthropophony). The bounding boxes were strategically positioned between presence segments to capture a wide range of sounds. Throughout the process, we made a conscious effort to exclude segments without any noise to enhance the precision of the final classifier. Furthermore, within the absence class, vocalizations of any species overlapping with the males' frequency range were annotated. Subsequently, Sonic Visualiser was utilized to validate the vocalization predictions made by the CNN and to eliminate any false‐positive or false‐negative predictions.

### 2.3 Machine learning methodology and process

We utilized a supervised learning approach to train our CNNs, employing code and methodology adapted from Dufourq et al. (2022). Initially, we annotated 135 audio segments from our 2020 recordings, amounting to 25 recordings from five individuals and 10 from another. Unfortunately, due to technical issues with one of the recorders, we had to exclude the annotations from this individual; their inclusion led to a decrease in the network's performance. Owing to limitations in computer hardware, we worked with a subset of the 125 annotated audio segments, specifically chosen to encompass a wide range of recordings across individuals, from various environments and weather conditions. Consequently, our 2020 training set comprised 8605 presence segments (augmented to 14,000; augmentation of data was done through time‐shifting of existing presence annotations) and 18,000 absence segments, with 14,000 being randomly sampled to ensure a balanced dataset. This preliminary network was then utilized to extract vocalizations from our 2019 recordings.

After annotating the recordings, we had to train our CNN, thus we searched for appropriate hyper‐parameters. After experimenting with over 40 different hyper‐parameter combinations, the values outlined in Table 2 yielded the best‐performing network. In the case of CNNs, fixed inputs are required, and given the considerable variation in the duration of multiple grunts—ranging from a fraction of a second to over 30 s—window lengths exceeding 4 s did not improve performance. Since grunt variations within a series of grunts are minimal, segments lasting longer than 4 s led to increased computation time without any performance gains. During our hyper‐parameter search, we also focused on determining the minimum and maximum call frequency values. Given that the fundamental frequency of reindeer grunts falls below 100 Hz, we set the minimum frequency to zero Hz while the maximum frequency was set to 4000 Hz. While formant frequencies of reindeer grunts tend to be indiscernible after 2500 Hz, setting the maximum frequency to 4000 Hz enhanced network performance, likely due to the provision of supplementary information that aided in distinguishing grunts from unintentional noises. However, setting the maximum frequency above 4000 Hz did not improve network performance and instead led to increased computation time.

**TABLE 2:** Pre‐processing hyper‐parameters used while training and analysing the rut vocalizations of reindeer from the Kutuharju field research station and the number of testing files used.

Parameters	Units
Low pass filter cut‐off (Hz)	1000
Downsampling rate (Hz)	8000
Nyquist rate (Hz)	4000
Segment duration (s)	4
Hann window length (samples)	1024
Spectrogram hop size (samples)	256
Number of spectrogram mel frequency bins	128
Spectrogram minimum frequency (Hz)	0
Spectrogram maximum frequency (Hz)	4000
Number of testing files (*n*)	12
Testing time (min)	1638.4

Four pre‐processing steps were then conducted to prepare the inputs for the CNNs, mirroring those detailed in Dufourq et al. (2022). First, a low‐pass filter was applied to each audio file to capture signals below its cut‐off frequency, which minimizes aliasing artifacts that can stem from downsampling. The filter's cut‐off frequency was determined based on the maximum frequency of the males' vocalizations in our presence annotations. Notably, the grunts of the males became indiscernible beyond 1000 Hz in Sonic Visualizer, with occasional louder calls detectable beyond this point, prompting the low pass filter's cut‐off to be set at 1000 Hz.

Subsequently, downsampling was performed on each audio file to enhance computational efficiency, given that higher frequencies were deemed excessive for the analysis. Sampling rates beyond 4000 Hz were found to offer no advantage in network performance and only increased computational demands. Therefore, the Nyquist rate was set at 4000 Hz, and the downsampling rate at twice that value (Dufourq et al., 2022).

Further, annotations for both classes were extracted using a sliding window approach. This involved segmenting each audio file into 4‐s windows initiated at the bounding box's start time, subsequently generating spectrograms that were then labelled accordingly (either presence or absence). This sliding window process continued until the window overlapped with the bounding box's end time, encompassing all bounding boxes across the entire dataset.

Lastly, the audio segments were transformed into two‐dimensional mel‐frequency spectrograms. The specific values associated with this transformation are outlined in Table 2.

During our training process, we evaluated various pre‐trained models detailed in Dufourq et al. (2022), and among them, the ResNet152V2 model (He et al., 2016) exhibited the best performance. We fine‐tuned both the feature extractor and the output layer to achieve the best possible model, although this adjustment led to increased computation time, it enhanced the network's performance. Similar to the approach outlined in Dufourq et al. (2022) and recognizing the necessity for pre‐trained models to have a three‐channel input (often aligning with the three channels in a colour image), we implemented the exponent method as described in the study. Specifically, we utilized the S^1^, S^3^ and S^5^ channels to generate our three spectrogram channels in line with the methodology outlined above.

To determine a spectrogram classification, the CNNs predicted two softmax outputs on each spectrogram within an entire testing file. The final classification (presence or absence) was determined according to the softmax output surpassing a value of 0.5. Each file was predicted by using a 4‐s sliding window approach. The window shifted 1 s at a time until an entire recording was processed.

After training the preliminary CNN, we used it to collect vocalizations from the 2019 recordings and verified the presence annotations manually to ensure accuracy and eliminate false positives. We also included absence annotations to provide the network with additional training data. We then used a subset of our 2020 annotations and all annotations from our 2019 recordings to train a final CNN. This final model was trained on a dataset of 10,778 presence and 11,546 absence annotations over 25 epochs. The final CNN was then used to detect reindeer vocalizations for the entire dataset. The number of files for each individual age group was as follows: 1.5‐year‐old, 11 files, 2.5‐year‐old, 58 files, 3.5‐year‐old 1, 22 files, 3.5‐year‐old 2, 53 files, 3.5‐year‐old 3, 21 files, 4.5‐year‐old, 28 files and 5.5‐year‐old, 93 files each file was 8 h long. It is worth noting that some files were <8 h due to recorder failure during the recording.

To evaluate the network's performance, we conducted tests using 12 audio files, none of which were used during the network's training phase. These files represented individuals from across the rut and various rutting behaviours. For instance, some files contained minimal vocalizations, while others featured over 250 instances. Among these recordings, eight were from the 2020 data, while the remaining four were from the 2019 data. To assess the network's performance, we used recall rate, precision, accuracy and F1 scores (Mesaros et al., 2016; Navarro et al., 2017; Equations S1–S4).

To train the networks, we utilized the packages listed in Table S1. The script was executed using Python 3, and the CNNs were implemented in Tensorflow 2 (Abadi et al., 2016). Each CNN underwent training across epochs employing the Adam optimizer with a batch size of 32 (Kingma & Ba, 2014). Subsequently, spectrograms were generated using the Librosa library (McFee et al., 2020). Model training and testing took place on a 2021 Apple MacBook Pro equipped with an Apple M1 Pro processor and 16 GB of LPDDR5 RAM, operating on MacOS Ventura 13.1.

### 2.4 Data analysis

We conducted statistical analysis in R v. 4.1.3 (R Core Team, 2018). To evaluate the hourly and daily patterns of our male reindeer over the 2019 and 2020 ruts, we used hierarchical generalized additive models (HGAMs, using the ‘gam’ function in the R package mgcv version 1.8–41; Pedersen et al., 2019; Wood, 2017).

The vocalizations predicted by our CNNs underwent visual and auditory verification, and their durations (in seconds) were aggregated by the hour of the day. These time intervals were based on the recorders' memory and cross‐checked using GPS data and audio from when the reindeer were released into the pen. Subsequently, the males were compared based on their estimated peak rut dates (denoted as ‘peak’ henceforth). These dates were estimated by averaging the spring calf births and then backdating using a gestation length of 221 days (Mysterud et al., 2009). The estimated peak dates for the 2020 and 2019 cohorts were October 6th and October 5th respectively.

Smoother terms were used to compare the patterns of the proportion of time spent grunting across days and hours in terms of individuals (Equation 1 [*i* indicates each variable is calculated for each individual]).

$$\text{Grunt proportion}\;g_{i}\left(t\right)\sim \text{Beta}\left(\mu_{i}\left(t\right),\theta \right)$$

(1) $$\text{logit}\left(\mu_{i}\left(t\right)\right)=f_{i}\left(\mathrm{Day}\right)+g_{i}\left(\text{Hour}\right)+\mathrm{re}\left(\text{Individual}\right)+\text{Stat}{\mathrm{us}}_{i}$$

The data were fitted using the beta regression family with a logit function and restricted maximum likelihood. To address zero values, an offset of 2.2 × 10^−14^ was used to prevent complete separation in the data. Status was modelled as a categorical variable with two levels: dominant and subdominant (Equation 1). Hourly patterns (Equation 1) were modelled using cyclic cubic regression splines, considering the repeating nature of 24‐h days. To provide a more general description of the pattern, 10 basis functions were used for the splines (Wood, 2017). Daily patterns (Equation 1) were fitted using thin plate regression splines, utilizing 22 basis functions to allow for flexibility in describing the pattern. Additionally, the smoothers fitted by individual (Equation 1) were considered as random effects to capture inter‐individual variability in the average frequency of vocalizations. Both temporal patterns were fitted using individual group‐level smoothers with varying wiggliness to describe the pattern of each reindeer (Pedersen et al., 2019). Finally, trends were fit using individual smoothers to describe the behaviour of the reindeer, and 95% confidence intervals were utilized to represent the uncertainty in the data (Wood, 2017).

The grunting activity of the reindeer was described by transforming proportions into time as a proportion of each hour. When reporting values, the mean and standard error (mean ± SE) were used. The fit and autocorrelation of the HGAM models were evaluated using the ‘appraise’ function in gratia (Simpson, 2021) and the ‘ACF’ function in mgcv (Wood, 2017).

## 3 RESULTS

### 3.1 Machine learning performance

The model's performance metrics varied, ranging from 93.6% to 97.8% depending on the specific metric. Although the accuracy stood at 93.6%, the precision, F1 score and recall rate were notably higher at 95.8%, 96.7% and 97.8% respectively (Appendix 1). However, the network exhibited an uneven distribution of false‐positive and false‐negative predictions, particularly originating from a subset of the files. Additionally, certain recorders showed a higher occurrence of false‐positive and false‐negative predictions across all their recordings compared to others. Overall, the network demonstrated higher‐than‐expected metrics when predicting upon individuals it was trained upon. Even when predicting upon individuals it was not trained on, the metrics often exceeded 80%, dropping below this standard only in scenarios where the recordings were heavily affected by wind or other consistently loud unintentional sounds (Appendix 1).

### 3.2 Rutting behaviour

**FIGURE 3:**
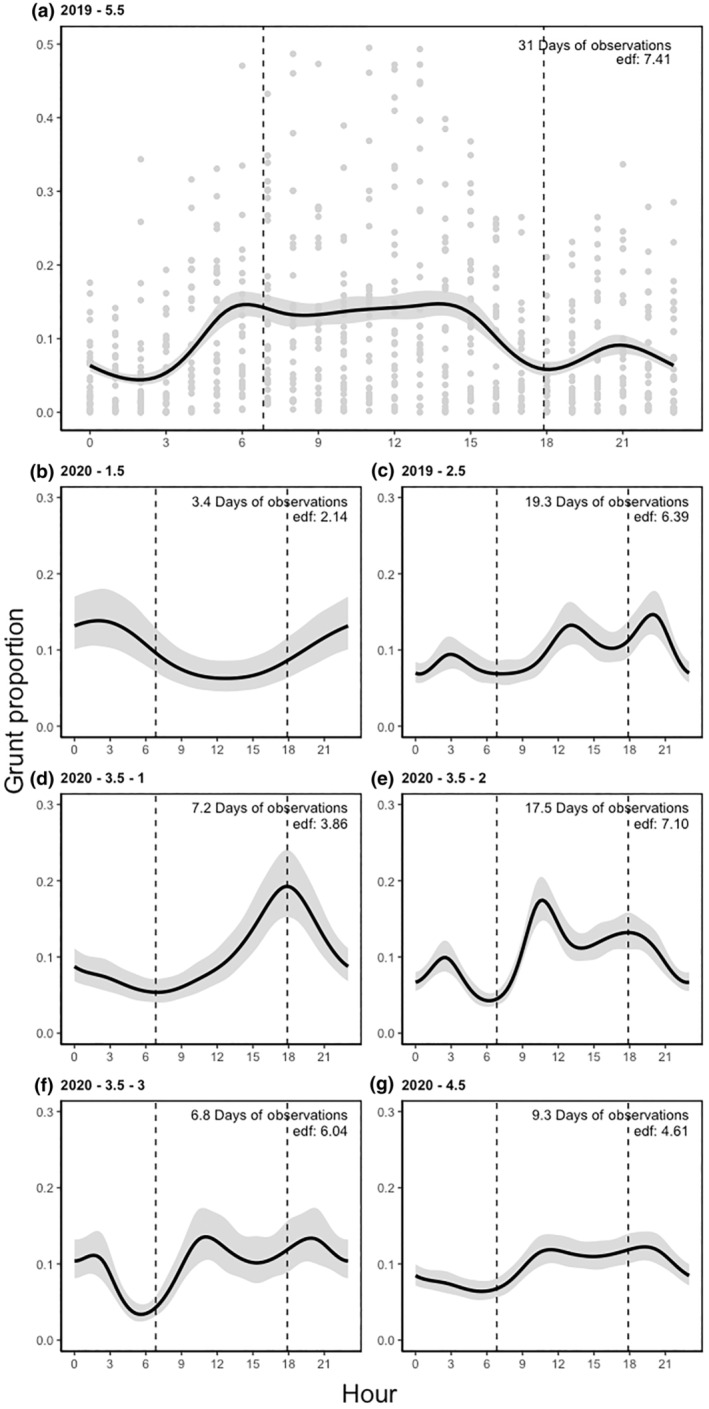
(a–g) Estimated smooth curves of the 24‐h rut patterns of seven reindeer according to their acoustic recordings represented as grunting behaviour taken as a proportion of time for each hour. (a) represents the estimated smooth curve of a 5.5‐year‐old and its complete data set to serve as an example for the data gathered. (b–g) represent six smooth curves of six individuals ranging from 1.5 years old to 4.5 years old. Black lines represent the mean, and shaded areas represent the 95% confidence interval. Models were fit using 10 basis functions, and edf represents effective degrees of freedom. Days of observation represent the number of days the smooth curves were estimated. 2019 and 2020 represent the year the data were gathered. 1.5–5.5 represent the age of the reindeer, and 1–3 represent different individuals of the same age class. The vertical dashed lines on the figure indicate the sunrise and sunset times in Kaamanen, Finland, on October 6th, at 06:50 and 17:53 respectively.

**FIGURE 4:**
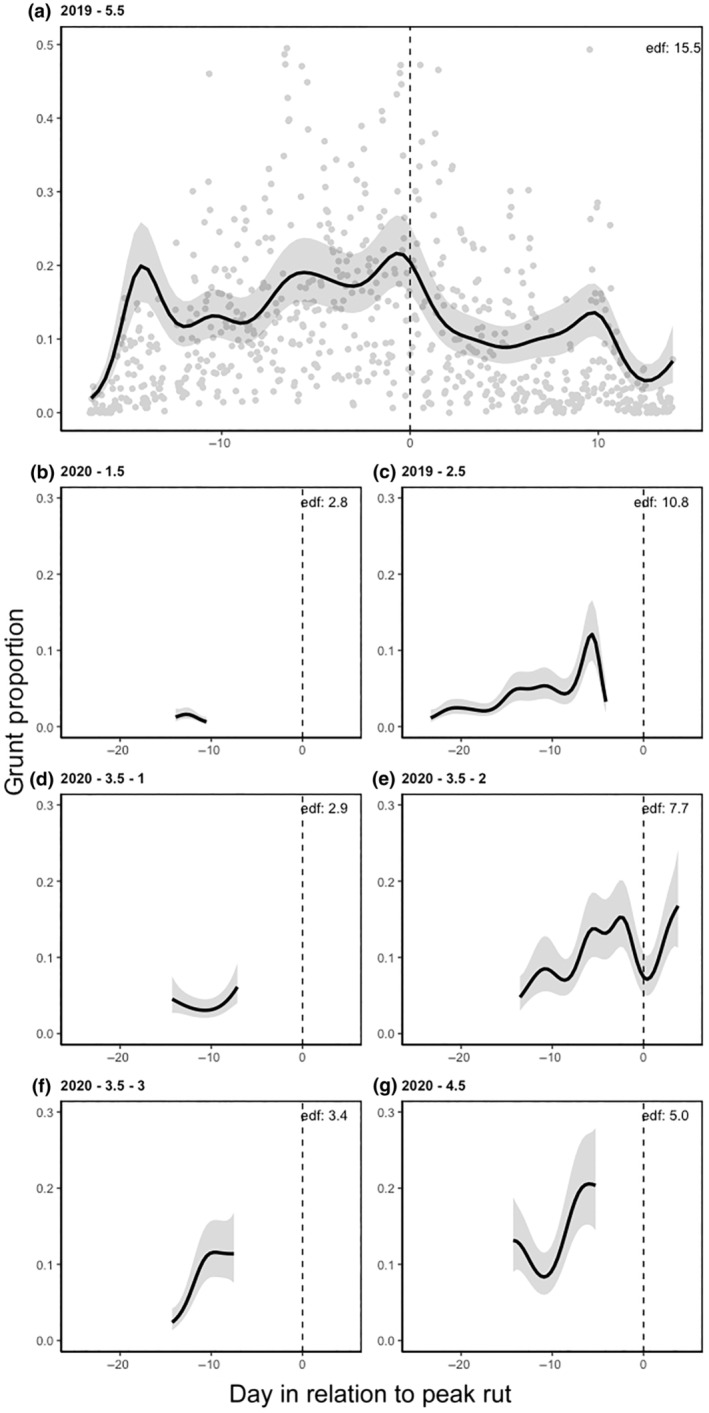
(a–g) Estimated smooth curves of the daily patterns of seven reindeer according to their acoustic recordings represented as grunting behaviour taken as a proportion of time for each hour. (a) represents the estimated smooth curve of a 5.5‐year‐old and its complete data set to serve as an example for the data gathered (note that the scale for this figure differs from the other six). (b–g) represent six smooth curves of six individuals ranging from 1.5 years old to 4.5 years old. Black lines represent the mean, shaded areas represent the 95% confidence interval, and the dotted vertical line represents the peak rutting day. Models were fit using 22 basis functions, and edf represents effective degrees of freedom. Days in relation to peak rut refer to the estimated peak rut date of that year's cohort. The peak ruts for the 2019 and 2020 males were estimated to be October 5th and October 6th respectively. 2019 and 2020 represent the year the data were gathered. 1.5–5.5 represent the age of the reindeer, and 1–3 represent different individuals of the same age class.

**FIGURE 5:**
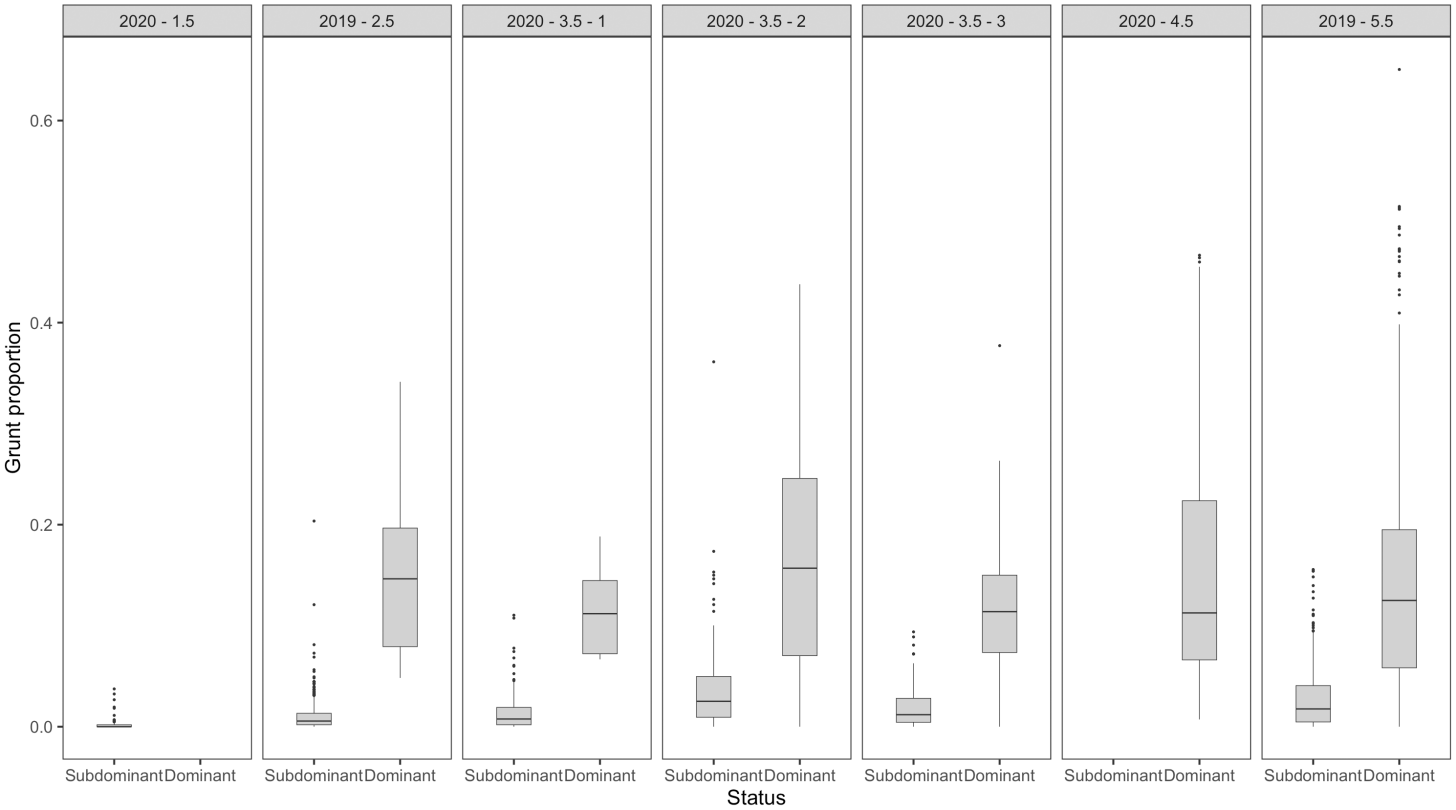
Comparison of hourly grunt proportions when an individual is in dominant or subdominant positions. 2019 and 2020 represent the year the data were gathered. 1.5–5.5 represent the age of the reindeer, and 1–3 represent different individuals of the same age class.

Between males, grunting activity varied by individual, with larger males grunting more (Table 1, Figures 3 and 4). When males were dominant and in control of a harem, they spent, on average, 14.2 ± 31.6 min per hour (HGAM; *z*‐value = −10.68, *p* < .001, *n* = 2213) grunting more than subdominant males. The model accounted for 83.2% of the deviance within the data with an adjusted *r*‐squared of .608. Generally, larger males grunted more compared to smaller males (Table 1, Figures 3 and 4). Within our male group, younger and older males exhibited distinct grunting patterns unique to their respective age groups (Table 1 and Figure 5). Notably, among the 3.5‐year‐old males, differences in age, weight and grunting patterns were more pronounced on an individual basis (Figure 5).

The 1.5‐ and 2.5‐year‐old males, weighing 83 and 96 kilograms, respectively, spent very little time in dominant positions, at 0% and 3.5% respectively. In contrast, the 4.5 and 5.5‐year‐old males, weighing 150 and 140 kilograms, spent the majority of their time in dominant positions, at 100% and 66.9% respectively. There were notable variations in the time spent vocalizing for the 3.5‐year‐old males, and their time spent in dominant positions, particularly considering their pre‐rut weights of 100, 135 and 155 kilograms. The lightest among them spent nearly no time in a dominant position, at 4.0%, while the other two spent roughly half their time in dominant positions, at 51.1% and 53.0% respectively. These findings are, however, tempered by uncertain data due to inconsistent recording lengths. Additionally, there was significant variety in the grunting patterns of the males, even when in dominant positions. When examining the grunting patterns of each male by status, there was a noticeable difference between the proportion of time spent grunting in dominant and subdominant positions, 8.85 ± 0.19 min versus 1.14 ± 0.05 (Wilcoxon rank sum test, *W* = 1,137,461, *p* < .001; Figure 5).

In the hourly data, the males exhibited several consistent patterns. They were generally more active during daylight hours, particularly from around 9:00 to 21:00 (Figure 3a,c–g), and their grunting decreased during the overnight hours, with the least grunting occurring just before dawn at approximately 06:00 (Figure 3a,c–g). The only inconsistency was observed in the hourly pattern of the 1.5‐year‐old male, who was consistently subdominant throughout the rut (Figure 3b).

The daily patterns among males (Figure 4) exhibited more variability. Larger males (Figure 4a,e–g) generally vocalized more than smaller males (Figure 4b–d). The oldest male (Figure 4a) was predominantly dominant throughout the rut and displayed increased grunting before the peak, followed by a decline afterward. In general, for dominant males, grunting rates tended to rise leading up to the peak and then decrease afterward (Figure 4). An exception was observed with the second 3.5‐year‐old male (Figure 4e), who demonstrated an increase in grunting rate about a week before the 2020 peak, followed by a decline for 2.5 days. The grunting rates of the two remaining males (Figure 4f,g) increased as they approached the peak, although due to premature recorder failure, limited information could be gathered from their audio recordings. Moreover, since there was substantial variation in grunting rates from 1 h to the next, there was notable variability in the males' daily rates, especially among dominant males as they approached the peak (e.g. Figure 4a). Overall, dominant males tended to vocalize more as the peak of the rut approached, compared to subdominant males (Figure 4).

## 4 DISCUSSION

The male reindeer's grunting patterns showed variability between males and across different hours and days during the rut. Older, heavier males tended to vocalize more and spend more time in dominant positions compared to younger, lighter males. However, this trend was not consistent among all males, as observed in the 3.5‐year‐old males. Dominant males were found to vocalize more than subdominant males, but there was still significant uncertainty as recorders captured varying grunting lengths. This suggests that not all males' grunting patterns were fully explored. Nevertheless, these findings are corroborated by the findings of previous research.

While collecting acoustics data provide some insight into reindeer rut behaviour, it only offers a partial explanation. It is important to approach these patterns with skepticism, as they may not fully capture the range of behaviours exhibited by reindeer. However, by combining both on‐animal acoustic recorders and machine learning applications, researchers can delve into behavioural questions that span greater spatial and temporal scales.

### 4.1 On‐animal acoustic recorders

Acoustic recorders have proven effective for non‐invasive behavioural research over extended periods and have captured otherwise undetectable sounds (e.g. Casoli et al., 2022; Insley et al., 2008; Lynch et al., 2013; Stowell et al., 2017; Studd et al., 2019, 2021; Thiebault et al., 2021; Wijers et al., 2018). For instance, Insley et al. (2008) used these devices to monitor the behaviour of northern fur seals (*Callorhinus ursinus*), a method we adapted for studying our males' grunting behaviour.

With the appropriate expertise, researchers can often differentiate between the audible behaviours of their species, although at times, ambiguous sounds require visual confirmation (Lynch et al., 2013; Wijers et al., 2018). For behavioural analysis, contextualizing acoustic data with observation is crucial. Furthermore, combined with other tools like bio‐loggers, as demonstrated by Wijers et al. (2018), acoustic recorders become more powerful, enabling refined behavioural insights.

Nevertheless, technical challenges such as high failure rates and the physical burdens of on‐animal recorders constrain their deployment mainly to larger species or shorter study periods (Casoli et al., 2022; Lynch et al., 2013; Stowell et al., 2017; Studd et al., 2019, 2021; Thiebault et al., 2021; Wijers et al., 2018). Consequently, prolonged studies on terrestrial species using these devices are scarce. Our study was limited to 1 month, and only one of our eight recorders lasted beyond 20 days. Addressing these practical issues could greatly enhance the utility of on‐animal acoustic recorders for ecological research.

### 4.2 Machine learning methodology and transfer learning

The increasing interest in machine learning methods within the field of ecology has led to improved performance. Dufourq et al. (2022) emphasized the power of acoustic classifiers as useful tools for researchers but noted the difficulty for machine learning novices in creating and training models.

Using transfer learning for CNN development, as highlighted by Dufourq et al. (2022), simplifies hyper‐parameter tuning and network design decisions. However, selecting a suitable pre‐trained model is a pivotal decision since the choice hinges on the model's original intended purpose. Therefore, opting for the right pre‐trained model can significantly impact the effectiveness of the model's new objective. Despite this, the use of transfer learning can expedite the initial development of classifiers.

Recent applications of machine learning for acoustic classification have demonstrated promising results. For instance, Studd et al. (2021) achieved F1 scores of 79 to 90% for Canadian lynx (*Lynx canadensis*) feeding event classifications using CNNs. Consequently, our ability to achieve performance metrics above 90% is promising. Failing to reach high‐performance levels can lead to the need for manual processing, which negates the impact of automated classifiers. Additionally, when the classification success rate is lower, there is a higher risk of missing out on valuable data trends due to mistakes in acoustic classification. As these methodologies advance and gain more traction among practitioners, their performance and ease of use will likely improve.

Despite its advantages, using machine learning in conjunction with on‐animal recorders presents challenges, particularly due to the animals' movements and the varying quality of recordings over time and across devices. As recorders can accumulate damage over time, the quality of recordings can also vary, affecting classifier performance. These changes in quality can generate additional false‐positive predictions. Studd et al. (2021) corroborated these findings when they used an automated event classification model to predict the hunting activity of Canadian lynx. While the process can be improved through manual verification and collection of annotations, practitioners must be aware of and address these limitations.

Although using a classifier can streamline data processing, the current level of reliability is not sufficient for full automation. Hence, predictions still require validation, and practitioners need to inspect classifier predictions to ensure accuracy. While the process accelerates bioacoustics data analysis, it is not yet entirely reliable without human validation. Nonetheless, the advantages of using machine learning are substantial.

### 4.3 Rut grunting patterns

The grunting patterns of various age and weight groups of semi‐domesticated reindeer were analysed in this study. While our findings are consistent with previous research, they offer a more detailed description of reindeer activity. Notably, this study is one of the first to depict reindeer's hourly grunting patterns through emitted sounds. Past research has shown that the age and weight of male reindeer are key predictors of their activity (Body et al., 2014; Espmark, 1964; Mossing & Damber, 1981; Tennenhouse et al., 2012). Specifically, older males generally spend more of their time grunting and are more active than younger males (Kojola, 1991; Mysterud et al., 2009; Skogland, 1989; Tennenhouse et al., 2012). However, the activity of older males diminishes during the late rut compared to the early and peak rut (Tennenhouse et al., 2012). This aligns with our study's findings, as grunting activity increased towards the peak and decreased thereafter. By the final weeks of the rut, older males depleted most of their energy reserves, impacting their ability to compete for access to females (Bobek et al., 1990; Leader‐Williams, 1980; Tennenhouse et al., 2012). This likely occurs because dominant males must defend their harems, affecting their ability to rest, while subdominant males can rest for longer. This decrease in grunting activity is observed in our 5.5‐year‐old male past the peak and is reflected in the birth of his offspring. Of the 14 offspring he sired, 13 were conceived in the 10 days preceding the 2019 peak.

Grunting patterns also shed light on the behaviours of smaller males. Younger males exhibit opportunistic behaviour, seizing opportunities to access females whenever possible, while older males display more polygynous behaviour, engage in more frequent fights, start the rut earlier, and are more active during the peak (Espmark, 1964; Mysterud et al., 2009; Tennenhouse et al., 2012). Younger males are generally more active during pre‐rut weeks than peak‐rut weeks (Tennenhouse et al., 2012). Observations in Dall sheep (*Ovis dalli*) suggest that young males increase mating activity when older males are scarce (Singer & Zeigenfuss, 2002). Younger males may struggle to time their reproductive efforts, unlike older males (Mysterud et al., 2008; Tennenhouse et al., 2012). This mating naivety in younger males may manifest as underdeveloped social rutting behaviour (Tennenhouse et al., 2012). Whether it is learned or situational, the timing of increased reproductive periods aligns with the males' vocal activity as well.

When males were dominant, they vocalized more frequently than when they were subdominant, indicating a correlation between their status and activity levels. While younger males occasionally attained dominance, they spent more time in subdominant positions compared to heavier males. However, there were instances where smaller males became dominant and larger males became subdominant, leading to significant changes in their grunting levels. The status of the males was a key determinant of their activity, with dominant males vocalizing more than subdominant males. Overall, dominant states were more prevalent among heavier, older males than younger, lighter males. The exact mechanisms behind these differences in activity are still not fully understood.

The hourly patterns of male reindeer were generally similar but exhibited slight differences. Typically, the early morning hours (00:00–06:00) saw the least grunting rates, with increased grunting rates during the morning and early afternoon, followed by a decrease in the later afternoon (15:00–18:00). Grunting rates then rose again towards dusk (19:00–21:00) before gradually decreasing. Notably, the 1.5‐year‐old male showed a different pattern, but due to limited data, his pattern should be viewed with caution. Unlike older, heavier males, the smaller males seemed more active towards dusk. Furthermore, our heavier males showed consistent grunting rates throughout the day. While most copulations in reindeer were said to occur during dawn and dusk in prior studies, our findings did not document extensive rutting behaviour during the early morning hours (Espmark, 1964). Similar activity patterns have been noted in other Cervidae species and goats, where mating activity peaks during the day and decreases during specific periods, correlating with times of increased temperature (Mellado et al., 2000; Zeng et al., 2011).

The grunting patterns of younger males exhibit an opportunistic approach, with increased rates later in the day, potentially after older males have expended much of their energy. However, this pattern varies among individuals, with only the increased rates later in the day consistently observed compared to older, heavier males. Furthermore, the overnight rates of all our males differ by individual, suggesting variability in their nocturnal behaviour. While previous remarks suggested that reindeer are comparatively inactive during the night (Espmark, 1964), our study found that males remained somewhat active overnight, even subdominant males. However, given the limitations of our data and the issues with our recorders, a complete depiction of the males' activity throughout the rut could not be achieved. Future research should focus on presenting a comprehensive understanding of each age class's activity.

Considering the limitations associated with on‐animal acoustic recorders, the grunting patterns of reindeer depicted in this study should be approached with caution. Vocal activity may not fully capture rutting behaviour, and machine learning networks may face challenges in categorizing the sounds emitted by a species. As technology continues to advance, these challenges may be addressed, enhancing the accuracy of future studies in this area.

## 5 CONCLUSION

Based on our findings, reindeer vocalizations are crucial for regulating rut behaviour. Our research suggests that males who vocalize more, tend to be dominant and have greater mating opportunities. This indicates the importance of vocalizations in sexual selection and harem management.

Reliability issues with on‐animal recorders present a significant challenge in using machine learning with this naïve technology. Thus, future endeavours should strive to rectify these ongoing issues. A prefabricated, commercially produced on‐animal recorder may be the best solution. Deploying commercial units would hopefully remove many of the reliability issues and increase the use of these technologies by additional practitioners, enabling researchers to deploy recorders for longer.

While machine learning shows promise, caution is needed when using on‐animal acoustic recorders. Both technologies offer substantial potential. Future research should focus on larger sample sizes to better understand reindeer rutting behaviour and optimize models for interpreting unintentional animal noises.

## AUTHOR CONTRIBUTIONS

**Alexander J. Boucher:** Conceptualization (equal); data curation (lead); formal analysis (lead); methodology (lead); validation (lead); visualization (equal); writing – original draft (lead); writing – review and editing (equal). **Robert B. Weladji:** Conceptualization (equal); funding acquisition (equal); investigation (equal); methodology (equal); supervision (lead); validation (equal); writing – original draft (supporting); writing – review and editing (supporting). **Øystein Holand:** Conceptualization (equal); funding acquisition (equal); supervision (supporting); writing – review and editing (supporting). **Jouko Kumpula:** Investigation (supporting); resources (supporting); writing – original draft (supporting); writing – review and editing (supporting).

## CONFLICT OF INTEREST STATEMENT

The authors have no competing interests to declare.

## Supporting information


Appendix S1

## Data Availability

The data are available at https://doi.org/10.5061/dryad.w6m905qx8 along with python script, data and the machine learning models.

## APPENDIX 1 The recall rate, precision, accuracy and F1 average values of two convolutional neural networks trained on a series of sound files containing reindeer vocalizations and unintentional sounds

### 1.1

**Table:**

Model	Audio file subset^a^	Recall rate	Precision	Accuracy	F1
Preliminary 2020 classifier	Trained (*n* = 8)	95.5	92.8	82.6	94.1
	Untrained (*n* = 4)	84.2	98.1	89.1	90.4
	Total (*n* = 12)	91.8	94.6	86.9	92.9
Final classifier	Total (*n* = 12)	97.8	95.8	93.6	96.7

^a^ The trained subset refers to eight testing files from individuals from the 2020 cohort, while the untrained subset refers to four testing files from the 2019 cohort (from two individuals, those audio files were not included in the training audio file batch). The total subset refers to the average values from a combination of the trained and untrained subsets (in the case of the combined model, the total subset represents the average of 12 testing files that came from individuals from both the 2019 and 2020 sampling periods, both of which were used to train this model). The 2020–50 Epoch Model was trained on 37 2.27‐h audio files from five individuals from the 2020 sample period over 50 epochs. This model was trained on 28,000 presence and absence segments. The combined – 25 Epoch Model was trained on 55, 2.27‐h audio files from seven individuals from the 2019 and 2020 sample periods. This model was trained on 10,778 presence segments and 11,546 absence segments. Testing files are audio samples that were not used during the training process.
