# Supplementary material for: Modelling reindeer rut activity using on‐animal acoustic recorders and machine learning
Source: Ecol Evol. 2024 Jun 25;14(6):e11479. doi: 10.1002/ece3.11479 (PMC11199844; doi:10.1002/ece3.11479)
Supplement: Supplementary file 1 — Appendix S1 [file ECE3-14-e11479-s001.docx]

**Supplementary material**

**Table S1**. The following Python packages were used to train the convolutional neural networks and predict the rutting audio of the male reindeer. The following files were run on Python 3.10.7 on a 2021 Apple Macbook Pro with an Apple M1 pro processor with 16 GB of unified LPDDR5 RAM running MacOS Ventura 13.1.

| **Python Package** | **Version** |
| --- | --- |
| **Jupyter** | 1.0.0 |
| **Keras** | 2.10.0 |
| **Librosa** | 0.9.2 |
| **Matplotlib** | 3.6.0 |
| **Numpy** | 1.23.3 |
| **Pandas** | 1.5.0 |
| **Pickle** | 0.7.5 |
| **Scikit-learn** | 1.1.2 |
| **Scipy** | 1.9.1 |
| **Soundfile** | 0.10.3.post1 |
| **Tensorflow 2** | 2.7.0 |
| **Yattag** | 1.14.0 |

**Equations S1-4.** The following four equations were used to measure our three networks' performance based on their predictions. True positives (TP) occur when a grunt presence is correctly predicted, false negatives (FN) occur when an absence of a grunt is predicted in a segment that contains a grunt, and false positives (FP) occur when a grunt is predicted in a segment when there is no grunt (Do Nascimento et al., 2021; Mesaros et al., 2016).

$$\left( S.1 \right) Recall rate or sensivity=\frac{TP}{TP+FN} \left( S.2 \right) Precision=\frac{TP}{TP+FP}$$

$$(S.3) Accuracy=\frac{TP}{TP+FP+FN}{\left( S. 4 \right) F}_{1}score=\frac{TP}{TP+\frac{1}{2}\left( FP+FN \right)}$$
